# Notes on Preparations for the Sick

**Published:** 1904-09

**Authors:** 


### Notes on Preparations for the Sick.

For the various analyses contained in this report we are indebted to Mr. Oliver C. M. Davis, B.Sc., A.I.C., of the University College, Bristol.

**Nutrico.**—The Maltico Food Company, Kingston Cross, Portsmouth.—A compound of cereal and farina with sterilised milk and a small proportion of organic iron compound. **Cremalto.**—A soluble combination of sterilised Devonshire cream, diastase, and malt sugars. **Leci Maltico.**

*Nutrico.*—This is an inexpensive food, designed for children and invalids. Analysis and microscopic examination show that the basis of the preparation is farinaceous, and the outer portions of the cereals used in its manufacture have not been entirely removed, the latter fact probably accounting for the high proportion of phosphates present in the ash. In addition, the presence of proteids was well indicated, so that the food may lay claim to its description as a true "protoplasmic" food.

*Cremalto.*—This substance is a preparation of extract of malt in combination with Devonshire cream. Chemical analysis shows that it contains maltose, but no added sugar. A good proportion of pleasant smelling, readily saponifiable fat was also extracted from it, confirming the fact that it is what the makers state it to be. It possesses the advantage of being very palatable, and should be serviceable where a fatty, nutrient food is required.

*Leci Maltico.*—This preparation is stated to differ from plain "Maltico" by the addition of a small proportion of lecithin. An examination shows that it possesses the usual characteristics of a well-malted food. Starch is entirely absent, but a large amount of proteids are present. The ash also gives reactions, showing the presence of iron and phosphorus.

---

**Savore.**—SAVORY AND MOORE, 143 New Bond Street, London.—The above preparation is described as a nutrient food, containing milk and cereal proteids. An analysis shows that its basis is starchy, but in addition it yields to cold water a good proportion of proteid matter. Phosphorus is also present in the ash. It should prove a valuable food for invalids where the use of farinaceous matter is not objectionable. It is already cooked, is appetising, and easily digested.

---

**Diabetic Foods.**—CALLARD & Co., Regent Street, London.—We have received a selection of these excellent preparations for the diabetic :—

Casoid Meal Bread, made from casein and starchless bran.
Casoid Rusks.
Casoid Biscuits and Lunch Biscuits.
Cocoanut Biscuits.
Ginger Biscuits.
Prolacto Biscuits. These contain 50 per cent. of albuminoids, and are free from starch and sugar.
Diabetic Marmalade, free from sugar.

Many so-called foods for the diabetic are not what they profess to be, and are very misleading not only to the patient but to the physician. Accordingly a careful examination of several of the above has been made in the University College Laboratory by Mr. Oliver C. M. Davis, B.Sc., A.I.C., who reports that they are worthy of special comment, inasmuch as they are almost absolutely starch free, and in some cases would not show the presence of starch when *chemically* examined. In all cases the amount of starch is limited to a very few scattered granules, so that great care has been exercised in their manufacture, and they are all that the makers claim them to be, being *virtually* free from starch and other carbo-hydrates. These preparations may be relied upon to be perfectly genuine, and worthy of all commendation.

With such foods at his command, and with so great a variety of palatable and nutritious kinds of bread and biscuits, there is no longer any valid excuse for the diabetic who declines to carry out a rigid diet of food free from starch and sugar. Whether such a rigid programme is necessary, and will conduce to the well-being of the patient, is for the physician to determine; but the difficulties in obtaining the necessary foods for the graver forms of diabetes no longer exist.

---

**Billon's Ovo-Lecithin Pills.**—London Depôt, 16 Water Lane, Great Tower Street, E.C.—Lecithin is a chemically-defined body generally extracted from the egg, but also found in brain, blood, milk, &c. The ovo-lecithin is the distearo-glycero-phosphine ether of choline. The combination has given good results in various forms of neurasthenia, also in pancreatic forms of diabetes and in the adynamia of the aged. In various forms of debility associated with anæmia and in tuberculosis it has been found to give good results. Each pill contains 5 centigrammes, and three to six may be given daily.

---

**Shredded Wheat. Triscuit..** — The Shredded Wheat Company, 6 and 8 Eastcheap, London, and The Natural Food Company, Niagara Falls, U.S.A.

The endeavour to utilise the whole of the wheat grain in the making of bread has led to the invention of the shredding process. The production of whole meal in the form of shreds which have been cooked and resolved to the consistence of a biscuit is the basis of various shredded products.

Shredded Wheat "Biscuit" and "Triscuit" are made by machinery direct from clean whole grains of steam-cooked wheat, without the husk or use of ferment or grease. Both are formed of threads of wheat laced and thoroughly cooked—the "Biscuit" being baked in Ferris Wheel ovens and the "Triscuit" baked by electricity.

Shredded Wheat "Biscuit" is a dainty little loaf or "pillow" of wheat, and provides a ready-cooked breakfast dish or basis for puddings and sweets.

"Triscuit," the electric cooked compact form, is used as bread, or biscuit, or toast, with butter, cheese, preserves, &c., and forms an excellent basis for savouries and sandwiches.

Both products are remarkably crisp and porous, and when eaten excite the flow of saliva and absorb the gastric juices, thus ensuring complete mastication and promoting digestion.

Chemical analysis shows a close resemblance between Shredded Wheat Biscuit and Triscuit and whole wheat meal, so that we have a porous and easily digested form of bread, prepared from wheat and nothing but wheat. Dietetically it is superior to the average bread, inasmuch as it contains a large proportion of soluble matters due to the presence of dextrinised starch. The biscuit keeps well, is not hygroscopic, and does not lose anything by evaporation. These products are a valuable addition to the dietetic armamentarium of the physician, and may be made the basis of a great variety of nutritious dishes.

---

"**Nektar**" **Non-Alcoholic Wines.**—REINHEIMER AND COMPANY, Surbiton, Surrey.

Samples of these wines were exhibited at the Oxford meeting, and attracted much attention. They are made at Worms on the Rhine, and have achieved a considerable reputation on the Continent during the last five years. They are excellent preparations of a pasteurised juice of the grape, apple, pear, bilberry, &c., and a considerable variety may be obtained—Hock, white or red, Tokay, Champagne; Bilberry, Pear or Apple Wine; also Bilberry and Apple Cider.

They are free from alcohol, and do not contain any artificial preservatives. Their food value is greater than that of fermented wines, inasmuch as they contain some fifteen to twenty-five per cent. of grape sugar in combination with organic acids and salts with albumen. Nektar Wines are especially indicated in all cases where the capacity for digesting and appropriating nutriment is greatly impaired, and in febrile maladies where the food should be in the fluid form. They are quickly and easily absorbed. The malic acid of Apple Nektar and the tartaric acid of Grape Nektar are cooling and refreshing, and tend to promote intestinal action, also to correct tendencies to constipation. Moreover, these organic acids are important as disinfectants in the alimentary canal, resisting the growth of disease germs and maintaining intestinal antisepsis. While in the system their salts become converted into carbonates, they impart alkalinity to the urine, and are on that account valuable in gouty states with a tendency to the deposition of acid urates. Owing to their laxative and diuretic properties, Nektar Wines are useful in all uric acid diseases, such as gout, rheumatism, &c. They have been found helpful in hæmorrhoidal affections, in hyperæmia of the liver, in chronic constipation, in cardiac diseases with a tendency to visceral congestion. The tendency to renal and hepatic concretions is often advantageously modified by the use of Nektar. It is prescribed with success in cases of abdominal plethora associated with a deposition of much superfluous fat. Its usefulness in cases of bronchial, gastric, and intestinal catarrh, and in anæmia, is well established. The happy combination of food qualities in these wines gives them a high range of utility, but no connoisseur will mistake them for the alcoholic products.

---

**Plasmon Tea.**—DIGESTIBLE TEA SYNDICATE COMPANY, 22 Fenchurch Street, London.

This variety of Plasmon is likely to be in much demand, inasmuch as it has the nutrient value of Plasmon in association with the stimulating proportions of a good tea. The amount of Plasmon in the tea is 10 per cent., and it is said that a large proportion of the tannin has been rendered innocuous. It is very convenient for use, and its flavour is excellent. Plasmon Tea is recommended to all who wish to have a good, digestible and nourishing tea; it is made in the ordinary way, but the presence of Plasmon gives the product a milky appearance when poured out.

---

**Helmitol. Citarin.**—The Bayer Company Limited, Elberfeld and London.

*Helmitol* is an improved and reinforced hexamethylene-tetramine (urotropin) which is being extensively employed as a urinary antiseptic. Experiments have shown that Helmitol liberates formaldehyde both in alkaline and in acid urine, and that 15 grains render the urine sterile for a longer period than does double that quantity of urotropin. Under the influence of Helmitol the urine becomes acid, which is of importance in ammoniacal fermentation, while it is not objectionable in acute conditions, since, owing to the analgesic action of the drug, any irritation is prevented without the necessity of administering alkalies. Hence Helmitol is indicated in acute and chronic affections of the urinary tract, such as cystitis, urethritis, pyelitis, &c. The average dose is 10 to 15 grains, two or three times daily, given preferably in water. Helmitol is very well tolerated by the system, and when given in moderate doses (*e.g.* 45 grains) no unsatisfactory effects as regards the gastro-intestinal canal, the circulation and nervous system, have as yet been observed. The internal administration of Helmitol in gonorrhœa may be well combined with injections of Protargol, which is considered by many authorities the most powerful gonococcicide.

*Citarin.*—Chemically, Citarin is anhydromethylene citrate of sodium. Its action depends upon the liberation of formaldehyde, which is set free in the blood and combines with uric acid, forming a combination which, as experiments have shown, is very soluble and easily eliminated. The other component of Citarin aids by reason of the fact that the salts of organic acids are converted into carbonates in the system, and thereby increase the alkalinity of the blood and its capacity of holding uric acid in solution. Experiments have shown that the elimination of the latter is increased by 35 per cent. after the administration of Citarin. Citarin is mainly indicated in that class of diseases in which an excessive accumulation of uric acid in the organism, and conditions directly resulting therefrom, are in question, particularly therefore all forms of gout. Experiments have shown it to be an excellent symptomatic remedy for all acute attacks of gout, and for the exacerbations occurring in the chronic stages of that disease; it surpasses even colchicum, and also has the additional advantages of being both innocuous and pleasant to take. The dose is in general 30 grains, from four to six times a day, for as long as the attack of goût lasts. It is well to begin treatment as early as possible and at the first sign of an attack. The simultaneous administration of aspirin has been recommended and proved beneficial. It is essential to success, however, that Citarin be taken *as early as possible*, viz. at the very first sign of an approaching attack. In this way it will often be possible to entirely suppress the attack by the aid of large doses of Citarin. As a prophylactic the administration of Citarin in uric acid diathesis may prove useful.

---

**Tabloids.**—Burroughs, Wellcome & Co., London.

Pilocarpine Nitrate.—The considerable variation in physiological action exhibited by the Galenical preparations of jaborandi has led to experiments which have proved that the full physiological effect of jaborandi is produced by pilocarpine, and, further, that the nitrate is the most suitable salt of the alkaloid for medicinal purposes.

Three Valerianates—Quinine, Iron and Zinc, āā gr. j.—A combination of the valerianates of quinine, iron and zinc has long been esteemed as an efficient tonic. "Tabloid" Three Valerianates enables this widely-useful compound to be employed in cases where the disadvantages of former methods of administration have rendered it objectionable.

Iron and Strychnine Phosphate, āā gr. j.—In this combination iron and strychnine are presented in the same dosage as in one fluid drachm of the official syrup of phosphate of iron with quinine and strychnine. It is useful in the case of those who cannot tolerate quinine.

Calomel and Opium—Calomel gr. j., Opium gr. $\frac{1}{2}$.—This well-known combination has the merit of antiquity, and in the tabloid form should be as useful as heretofore.

Calomel and Sodium Bicarbonate, Grey Powder and Sodium Bicarbonate.—These tabloids are prepared in two strengths: half a grain and one grain of the mercurial with two and half grains and five grains of the sodium salt respectively. They effectually dispose of the disadvantages associated with the administration of these drugs in the form of powders.

Soloid Mercuric Potassium Iodide, gr. 4.37.—The powerful antiseptic properties of mercury biniodide are well known, and the "Soloid" products of Mercuric Potassium Iodide, hitherto issued, have been very much appreciated and widely prescribed by medical men as a means of securing the effects of mercury biniodide in solution. In order to extend the usefulness of this agent, "Soloid" Mercuric Potassium Iodide, gr. 4.37 (0.283 gm.), is now issued. One dissolved in ten ounces of water forms a solution of 1 in 1,000 (frequently known as mercury biniodide solution). The colour of "Soloid" Mercuric Potassium Iodide is due to a harmless ingredient added as a safeguard against errors.

### List of "Soloid" Preparations.

"Soloid" Mercuric Potassium Iodide, gr. 1.75 (0.113 gm.). Issued in tubes of 25 and bottles of 100.

"Soloid" Mercuric Potassium Iodide, gr. 4.37 (0.283 gm.). Issued in bottles of 25 and 100.

"Soloid" Mercuric Potassium Iodide, gr. 8.75 (0.567 gm.). Issued in bottles of 25 and 100.

"Tabloid" Ophthalmic Zinc Sulphate, gr. $\frac{1}{250}$ (0.00026 gm.), and Cocaine Hydrochloride, gr. $\frac{1}{20}$ (0.0032 gm.).—This combination of zinc sulphate with cocaine hydrochloride is now issued. It will be found useful when it is desired to prescribe an astringent painlessly in ophthalmic practice. "Tabloid" ophthalmic products are immediately soluble on the conjunctiva, and are rapid in their action.

"Tabloid" Glycerophosphates Compound, dr. $\frac{1}{2}$ (1.8 c.c.).—"Tabloid" Glycerophosphates Compound possesses many advantages over the ordinary compound syrup of glycerophosphates which have been so extensively prescribed during the past few years in the treatment of enfeebled conditions of the nervous system. Each "tabloid" product contains the combined glycerophosphates of calcium, sodium, potassium, magnesium and iron, with pepsin, diastase, ignatia amara, and kola, equivalent to half a fluid drachm of syrup of glycerophosphates. All risk of deterioration by evaporation, crystallisation, or oxidation, so frequently associated with ordinary syrups, is entirely avoided when "tabloid" products are prescribed.

---

**Ext. Radicis Pernionis.**—Arthur & Co., Ogle Street, London, W.

This concentrated tincture of the *Tamus communis* is said to be a valuable hæmostatic, and is guaranteed to relieve the itching of chilblains almost immediately.

**Aquaform,** Di-oxy-methylene-hydrate.—This solution is said to be very efficient in relieving perspiring hands.

**Tanno-Pumilio.**—This compound of digallic acid with the essential oil of the pumilio pine is reported to give excellent results as a local application in cases of excessive perspiration of the feet. It also allays the irritation of herpes zoster.

---

**Dr. R. B. Waite's Local Anæsthetic.**—The Dental Manufacturing Company, London.

This aqueous solution of many drugs is very useful in dental operations, and as it is not a secret preparation we are able to speak in its favour. It contains less than 1 per cent. of cocaine, so that when using this solution one is able to obtain good results whilst introducing only a very small dose of the drug into the system. For the painless extraction, two or three minims should be injected into the gum on each side of the tooth. It is also useful for deadening the gum before applying the rubber dam and silk prior to the filling of teeth. It can also be used before separating front teeth when more room is required for the operation of filling.

**Adrenalin,** for dental purposes.—Adrenalin is a styptic, and is used by dentists in operations on the living pulp of a tooth. In the preparation of a cavity for filling, in a carious tooth, the pulp is often exposed. If it is decided to kill the nerve, a little of the fluid should be applied to the bleeding surface, when the pulp immediately shrinks away, and the dentist is then able to enlarge the opening without giving any pain. A crystal of cocaine can now be applied, and then a piece of soft dental base rubber may be pressed on the pulp. The pressure, as well as the action of the adrenalin, forces all the blood out of the pulp, which pulp can then be extracted and removed without pain to the patient.

---

**Glyco-Thymoline,** an alkaline, antiseptic, deodorising, and non-irritating solution.—Kress & Owen, New York.

It owes its value to the fact that it is a non-irritating antiseptic. For this reason it is useful for washing out internal cavities and passages, such as the nose, bladder, &c. A solution of half an ounce or more to the pint of warm water can be used in nasal catarrh with at least temporary benefit. A stronger solution (20 or 25 per cent.) makes an excellent mouth wash or gargle in foul conditions of the pharynx, gums, &c. It has been found distinctly useful in cases of cystitis, especially in elderly men. In all these and in other conditions its antiseptic action is not very powerful, but it is always a safe drug to employ, and it usually gives good results.

